# Exploring Usability of a Clinical Decision Support System for Cancer Care: A User‐Centered Study

**DOI:** 10.1002/cnr2.70173

**Published:** 2025-03-20

**Authors:** Darya Chyzhyk, Maddi Arregi, Maria Errazquin, Ainhoa Ariceta, Macarena Sevilla, Roberto Álvarez, Maider Alberich Inchausti

**Affiliations:** ^1^ Naru San Sebastian Spain

**Keywords:** adverse events, clinical decision support system, digital health intervention, eHealth, ePROMs, oncology, usability test

## Abstract

**Background:**

Adverse effects are a common burden for cancer patients, impacting their well‐being and diminishing their quality of life. Therefore, it is essential to have a clinical decision support system that can proactively monitor patient progress to prevent and manage complications.

**Aims:**

This research aims to thoroughly test the usability and user‐friendliness of a medical device designed for managing adverse events for cancer patients and healthcare professionals (HCPs). The study seeks to assess how well the device meets both patients' and HCPs' needs in real‐world scenarios.

**Methods and Results:**

The study used a multi‐method approach to obtain a comprehensive understanding of participants experience and objective measure of usability. The testing was conducted with a diverse group of participants of six patients and six HCPs. Analysis included a descriptive summary of the demographic data, scenario completion rates, System Usability Scale (SUS) questionnaire score, and qualitative feedback from users. All participants successfully completed 100% of the activities, indicating a high level of understanding and usability across both user groups. Only two out of six patients encountered errors during the login activities, but these errors were unrelated to product safety. The obtained SUS score is in the 90th percentile for both user groups, classifying the device as grade A and highlighting its superior usability. Patients and HCPs found the interface intuitive and expressed an interest in incorporating the application into their daily routines and would recommend the application to others.

**Conclusion:**

The assessed digital health medical device demonstrates excellent usability, safety, and ease of use for oncology patients and HCPs. Based on the received constructive feedback, minor improvements were identified for further refinement of the application that do not affect either its intended functionality or the overall functioning of the tool. Future work will focus on implementing these improvements and conducting further usability studies in clinical environments.

**Abbreviations**
AE: adverse events
ASQ: After Scenario Questionnaire
CGS: Curved Grading Scale
HCPs: healthcare professionals
PROs: patient‐reported outcomes
PROMs: patient‐reported outcome measures
PSSUQ: post‐study system usability questionnaire
QoL: quality of life
SAEs: severe adverse events
SUS: System Usability Scale
UMUX: usability metric for user experience

## 1 Introduction

Around 45% of all oncology patients suffer from severe adverse events (SAEs) related to treatment [1], which often are translated into unplanned hospitalizations, treatment interruptions, or treatment dose reductions. On top of that, symptoms managed inappropriately are one of the main causes of avoidable emergency room visits [2]. It has been proven that a routine assessment of patient‐reported outcomes (PROs) in the care of patients with cancer improves symptom management and patient‐clinician communication [3] and reduces anxiety among care partners [4], among others. Multiple studies show that a systematic monitoring of patients through PROs improves patient‐doctor communication, the understanding of the doctor regarding the symptoms suffered by the patient, symptom management, patient satisfaction, quality of life, and overall survival [2].

In the last decade, the number of electronic systems that have been developed to provide support to patients during and after cancer treatment through the use of patient‐reported outcome measures (PROMs) to evaluate symptoms remotely has increased significantly [5]. Electronic web‐based PRO capture tools or ePROs allow more timely and place‐flexible reporting than on‐site reporting or other traditional methods, which makes them more likely to enable better monitoring of changes in symptoms or quality of life and have a great potential to improve patient care by providing a comprehensive documentation of symptoms [6] and side effects [7]. In addition, ePRO systems can be coupled with an urgency algorithm that sends an alert to the care unit regarding severe or altered symptoms of a certain patient. This enables a rapid reaction from healthcare professionals (HCPs) and a rapid treatment of relevant clinical or medical events [8]. As a result, it has been seen that the use of the ePRO platform for symptom management follow‐ups in cancer patients is timesaving, energy saving, and effective [9]. It has been shown that using an ePRO platform for symptom management follow‐ups in cancer patients is timesaving, energy‐saving, and effective. To help expand these benefits across health systems, numerous commercial ePRO systems, such as Step Proactive, have emerged on the market.

**FIGURE 1:**
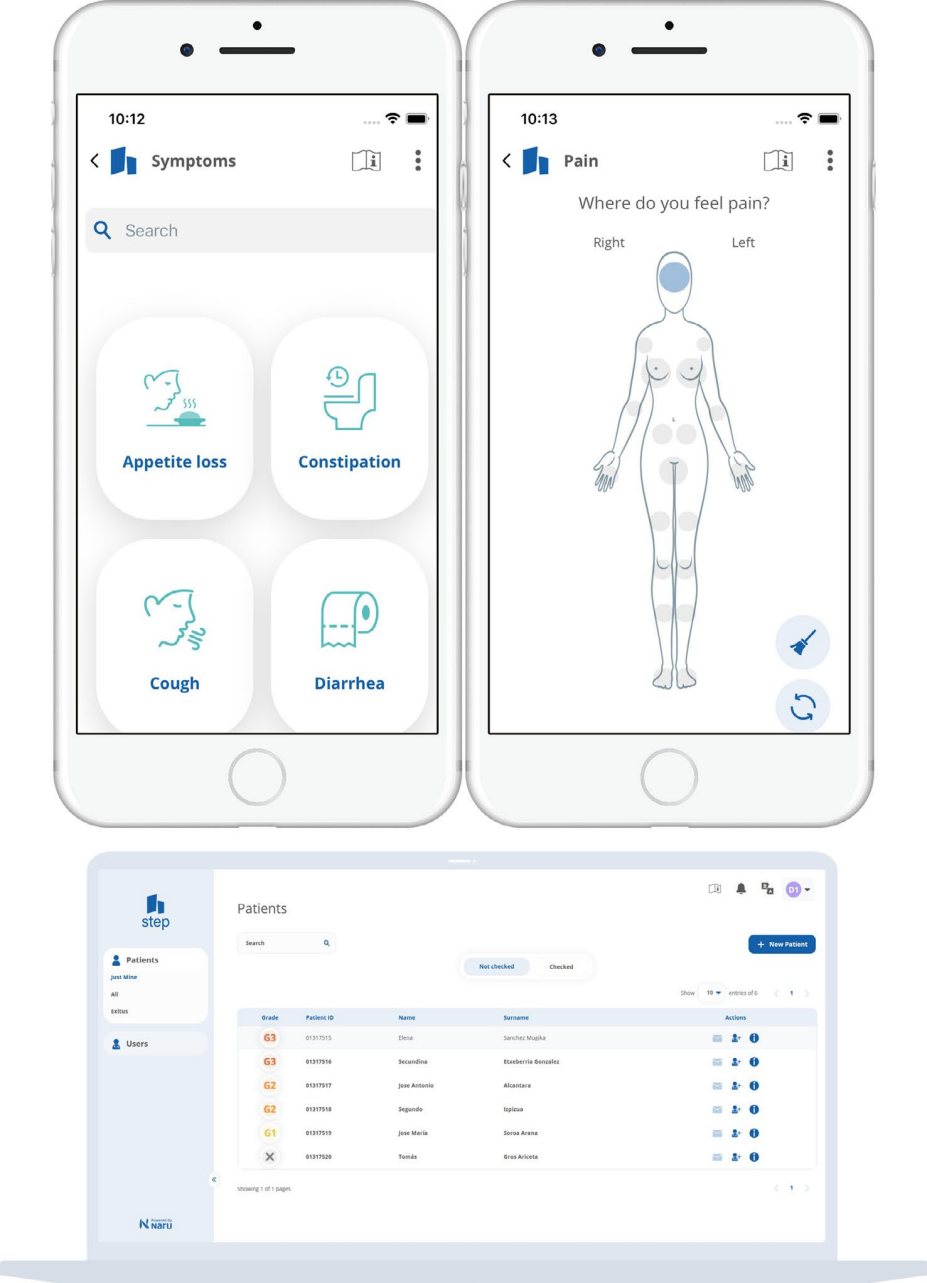
Step proactive interfaces. The first set of screenshots represents the patient user interface. The first screenshot displays various symptoms that patients can report, while the second showcases how pain reporting is done with gender‐specific adjustments. The third screenshot illustrates the interface used by HCPs. This screen capture demonstrates a visualization feature that allows HCPs to prioritize patients based on the severity of the patient reported data.

Step Proactive (Figure 1), which is a medical device software design developed and owned by Naru according to Regulation MDR 2017/745, is an adverse events (AE) management software that assists HCPs and adult patients without cognitive impairments in better managing AEs during and after cancer treatment. It enhances clinical follow‐up by notifying medical teams about symptoms beyond acceptable levels so that therapies can be adjusted or prioritized if further check‐up or diagnosis should be performed. The software facilitates proactive symptom management [10], early complication detection, and improved patient‐clinician communication [11, 12], leading to reduced emergency visits [13, 14], sustained treatment timelines, enhanced quality of life (QoL) [10], and contributes to patients living longer [15].

Due to their potential benefits to their users, digital health and mobile medical apps have shown great promise in transforming healthcare, but their adoption has sometimes been unsatisfactory in clinical environments and their retention is one of their most significant issues. Therefore, to potentially avoid these issues, the framework for multidimensional assessment of digital health products based on their lifecycle includes usability testing as one of the recommended dimensions [16]. Consequently, usability plays a critical role in the development and refinement of applications, particularly within the healthcare domain. Indeed, a usability test is one of the best ways to ensure that a product meets the user's needs. According to the international standard IEC 62366‐1:2015 [17], usability is defined as the capacity of the product to be effectively used by specific users in order to achieve specific objectives with efficiency and satisfaction. For companies operating in the healthcare sector, there is a growing motivation for properly designed and conducted usability tests of these digital health and mobile medical apps [18, 19, 20, 21].

Healthcare products directly impact patient well‐being and the efficiency of the hospital workflow [22]. Through usability testing, companies can identify key issues that may negatively impact how planned users interact with systems, essentially leading to better patient care and more efficient healthcare processes. Usability testing not only aligns with regulatory requirements and quality standards but also empowers companies with valuable insights to refine their applications, reduce potential risks, and align with the needs and preferences of the user. In the competitive technology industry, investing in usability testing becomes a strategic action for building customer trust and gaining overall quality of service.

In particular, this work shows a comprehensive usability test of the different Step Proactive interfaces. Individual interviews with 12 end‐users (6 for the interface of the patient version and 6 for the interface of the HCPs version) were conducted according to the requirements of IEC 62366 to assess the usability and clarity of the Step Proactive design. A multi‐method approach was used to evaluate Step Proactive's usability in order to identify potential use errors and developments to refine Step Proactive. This method comprised (1) user observation during the design task to gather qualitative data on user interactions and behaviors and (2) System usability approach to provide a complete understanding of subjective experiences and objective usability measures.

User retention and satisfactory adoption of Step Proactive are key for the product to be able to offer its potential benefits to patients. Usability was considered from the early stages of the Step Proactive lifecycle. Promising results in usability testing are the first step of a multidimensional assessment to ensure user retention and satisfactory adoption of Step Proactive. The aim of this work is to describe the process and assessment of the usability and user‐friendliness of the Step Proactive medical device, verified through user testing.

## 2 Methods

### 2.1 Step Proactive

Step Proactive is an intelligent patient‐centric cloud‐based system that enables a proactive care for patients, medical teams, and institutions. Step Proactive is able to identify and classify early signs of serious complications and prioritize patients if reported symptoms are outside an acceptable range, identifying patients at risk and improving the productivity of HCPs. This, together with the notifications generated by the algorithm, allows the healthcare team to make proactive and informed decisions when prioritizing clinical follow‐up actions and/or adjusting oncology treatment to avoid the development of pathologies associated with oncology treatment. Based on the classification of the information reported in real time by patients, the algorithm is able to generate standardized and clinically validated recommendations, thus personalizing patient care.

**FIGURE 2:**
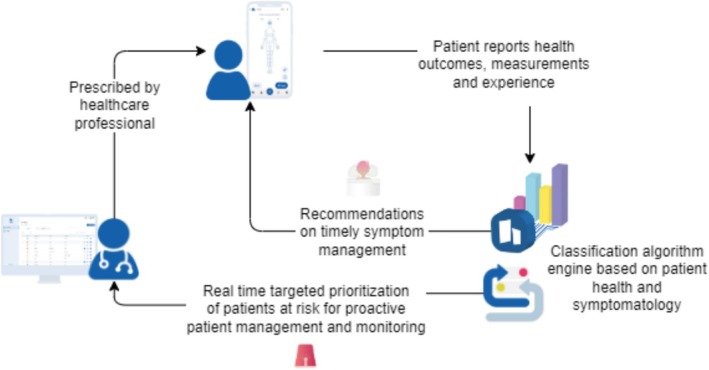
Workflow of step proactive. Patients with cancer, prescribed by clinicians, register on the application, and report their symptoms and adverse events using the step proactive interface. Patient‐reported outcome measures data is processed through a classification algorithm engine based on patient health and symptoms. The system provides recommendations related to symptom relief (e.g., diet, habits, exercise) to patients through the patient interface but in no case clinical recommendations (e.g., prescription of medication for symptom relief), while also delivering prioritized data on at‐risk patients to the interface for HCPs.

Step Proactive counts with two different interfaces, Figure 2: patient interface, through which patients may report valuable information about their health and receive timely advice about symptom management, and HCPs' interface, through which teams receive real‐time notifications concerning patient's status, detection of patients at risk, visual patient prioritization, and a holistic view of patients' health status involving both clinical outcomes and patient experience.

Step Proactive development has been performed in compliance with Medical Device Regulation (MDR) 2017/745 [23, 24] and IEC 62304. MDR is a comprehensive regulatory framework established by the European Union for medical devices. It contains stringent requirements that aim to ensure the safety, performance, and quality of medical devices placed on the European Union market. Compliance with MDR is mandatory for all medical devices, including software applications.

In the context of product development and design, we followed IEC 62304 Medical Device Software—Software Life Cycle Processes [25], an international standard that provides a framework for medical device software lifecycle processes. This includes conducting risk assessments, clinical evaluations, and usability studies to demonstrate the safety and user‐friendliness of the medical device [26].

Furthermore, IEC 62304 requires a medical device software development organization to implement a quality management system to show the ability to provide medical device software that aligns with regulatory requirements and customer expectations. This standard is ISO 13485 Medical Devices—Quality Management Systems—Requirements for Regulatory Purposes [27]. Our development process adheres to the standard design and development cycle defined by ISO 13485. In 2023, Naru achieved ISO 13485 certification, showcasing a commitment to developing quality, safe technology with the highest performance and satisfaction for our end‐users and customers.

**FIGURE 3:**
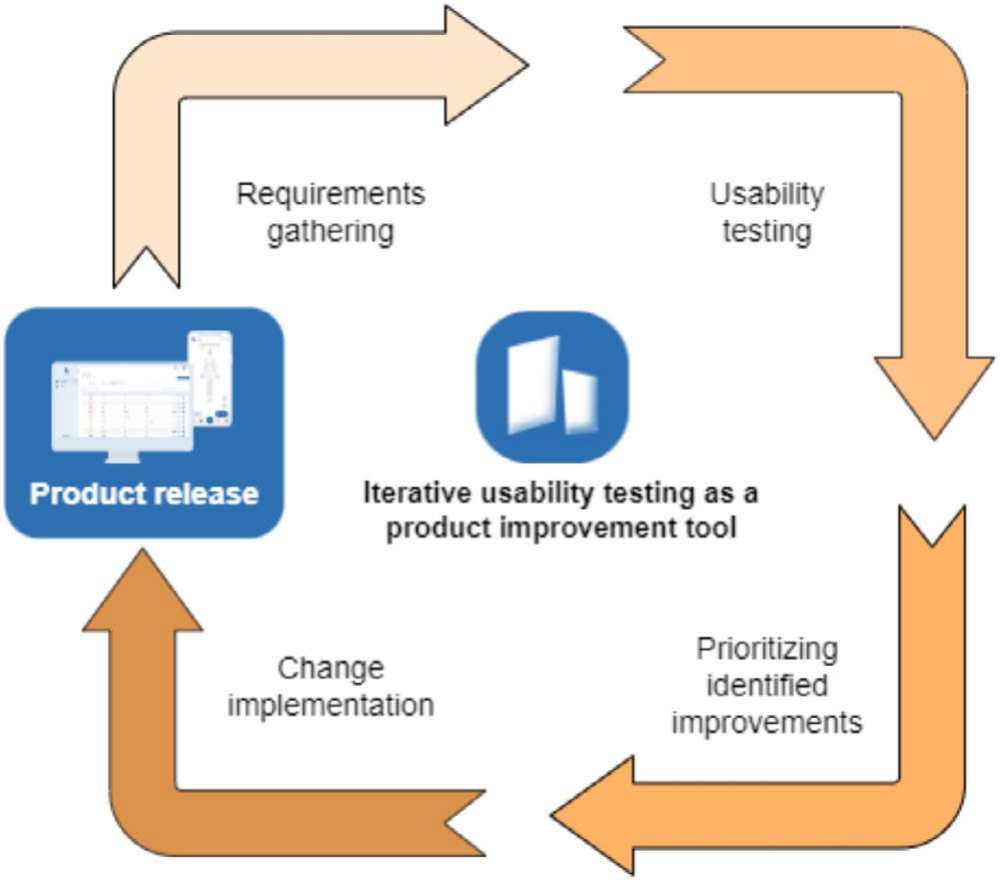
Iterative usability testing as a product improvement tool. This is a visual representation for the systematic process of iterative usability testing that we practice in Naru for all our products including step proactive, which serves as a powerful tool for continuous product enhancement. The circle begins with the requirement gathering and usability testing to identify areas for improvement. Then the team prioritizes the identified changes and implements them, leading to new features release. The cycle then repeats, ensuring correct refinement and evolution of the product and higher user satisfaction.

Usability testing is an iterative process, with refinements and improvements being made based on participant feedback. Figure 3 shows Naru iterative usability testing as a continuous product improvement tool. With user feedback, design and development teams gather evidence to validate incremental product improvements. This cyclic methodology ensures user involvement and ultimately ensures user‐centric approaches that benefit the overall user experience.

A smaller sample allows for quicker iterations, enabling more rapid improvements to the system based on user insights. As part of this cyclical work, this study shows one of Naru's usability testing iterations, focusing on obtaining quick and actionable feedback. This is the process that Naru has implemented in order to identify and minimize user errors and, above all, ensure safety for users in accordance with IEC 62366‐1:2015 [17].

### 2.2 Study Design

**FIGURE 4:**
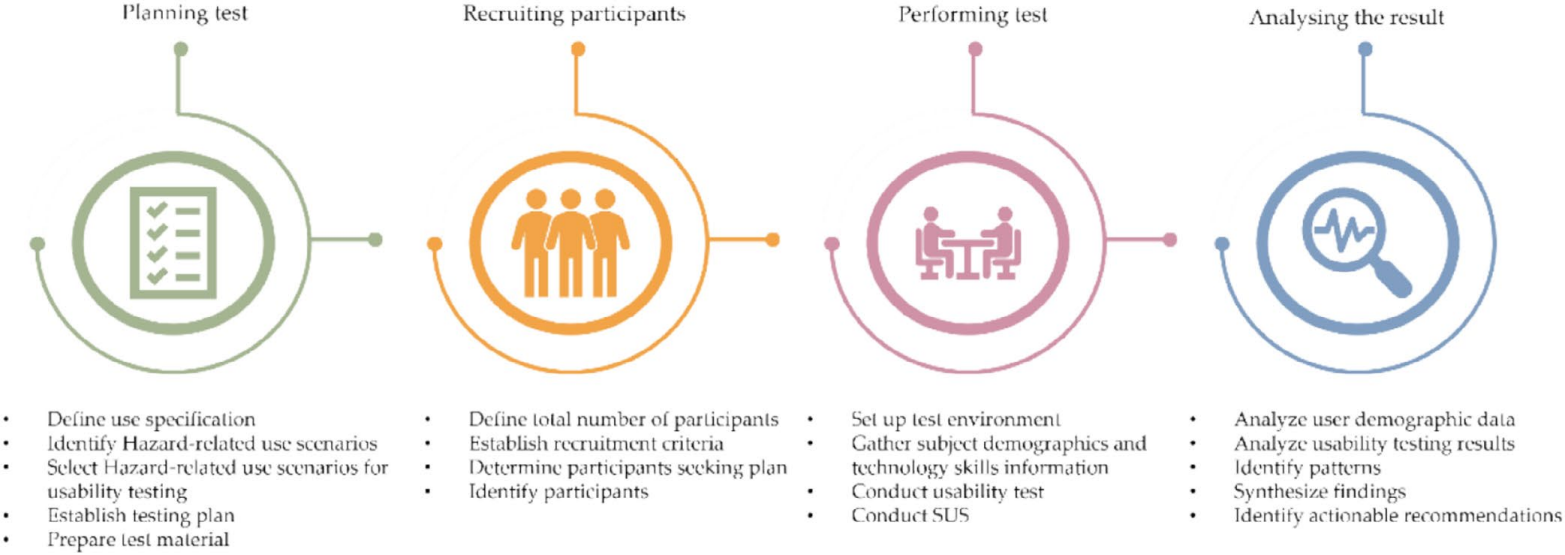
The usability study design process. This process is composed of four main phases:(1) planning test, (2) recruiting participants, (3) performing test and (4) analysis of the result.

To assess the usability of Step Proactive design, a multi‐method approach was employed. This involved two main methods: (1) User observation during the design task allowed for gathering qualitative data by closely examining how users interacted with the system [6]. By observing their actions and behaviors in real time, valuable insights were gained into areas where improvements could be made. (2) In addition to user observation, a standardized quantitative System Usability Scale (SUS) [28] was administered. By combining these two methods, it is possible to obtain a comprehensive understanding of both the subjective experiences and objective measures of usability. Figure 4 shows the total stages of the usability test involved in Step Proactive.

### 2.3 Planning Test

This stage includes the preparation of the usability test of Step Proactive. A plan for the development of the sessions was established to define the schedule of the sessions, human and material resources needed, and the physical environment in which the sessions will take place.

These preparations include the identification of potential risks [29], as well as the definition and selection of scenarios accompanied by detailed tasks and creating a protocol with clear instructions and guidelines for participants to follow. The quantity of scenarios for user testing depends on the number and type of detected hazards and possible outcomes after trying the product.

Another critical element is developing realistic scenarios [30] aligned with desired outcomes for evaluating the product's usability objectively as usability testing typically takes place in a controlled setting. Supplementing these scenarios with specific tasks will provide participants with clear instructions on what actions they should perform while using the application or system. Usability tests were to be conducted following the cognitive walkthrough protocol [31], which consists of a walkthrough of a scenario that could occur while performing the user's daily tasks, commenting on the use of the interface.

To ensure usability, objective evaluation covered realistic interaction with the tool; a total of 52 activities were designed and comprised in different scenarios, 16 specifically designed for patients and 36 for HCPs. Activities were grouped in seven scenarios designed for patients and 11 scenarios designed for HCPs. These scenarios were developed following a logic itinerary that a user may complete while doing their daily tasks on Step Proactive and provided context and purpose for the tasks performed by the participants in usability testing.

For each scenario's task, the end user, either patient or HCP, received specific instructions to perform meaningful and representative use of the tool. Scenarios covered interactions with essential features such asapplication login, symptomatology reporting, or patient prioritization. In Table 1 a list of the defined scenarios and the description of the representative interaction tasks performed during the usability evaluation are described.

**TABLE 1:** Usability testing scenarios and description of the tasks.

End user	Scenario no.	Essential feature dimensions	Tasks description
Patient	1	Product familiarization	Identification of warnings and use contraindications
Patient	2	Product familiarization	Access to instructions of use
Patient	3	Real‐time patient reported outcomes	Real‐time information and measurements
Patient	4	Real‐time patient reported outcomes	Symptom reporting
Patient	5	Real‐time patient reported outcomes	Real‐time information editing
Patient	6	Real‐time patient reported outcomes	Clinical information timeline checking
Patient	7	Real‐time patient reported outcomes	Quality of life measurements reporting
HCP	8	Product familiarization	Identification of warnings and use contraindications
HCP	9	Product familiarization	Access to instructions of use
HCP	10	Patient risk assessment and prioritization	Interaction with system's notifications
HCP	11	Patient risk assessment and prioritization	Patient prioritization interpretation
HCP	12	Patient health assessment	Patient status checking
HCP	13	Patient health assessment	Patient real‐time information checking
HCP	14	Patient health assessment	Patient evolution trend checking
HCP	15	Product familiarization	HCP user panel management
HCP	16	Product familiarization	Patient user panel management
HCP	17	Product familiarization	HCP user registration
HCP	18	Product familiarization	Patient user registration

All these scenarios contain safety‐relevant activities and activities related to the evaluation of the ease of use of Step Proactive. The tasks of each scenario are designed to uncover potential usability problems that could compromise user safety. These activities are important because they directly impact user safety and well‐being. Also, they are focused on understanding how easy it's for users to navigate the interface and understand the product features. The relevance of these activities lies in the fact that ease of use directly impacts user satisfaction, user engagement, and effectiveness [32, 33, 34]. By evaluating ease of use, it's possible to identify usability issues that reduce user efficiency or lead to errors. For example, in scenario 4, participants were asked to navigate through different sections of the product and report previously defined symptoms in real‐time. Instructors and monitors observed if participants could find what they were looking for, if they faced any navigation challenges or confusion, and if they completed the task.

### 2.4 Recruiting the Participants

In this stage, both the target user population and the total number of participants to recruit to assess Step Proactive's easeofuse and design clarity were defined. The assessment of these parameters with a relevant sample of Step Proactive's intended users is key. Namely, Step Proactive does not contemplate any restrictions regarding the technological skills of its intended users other than being able to use a device with internet access. However, patients who have any physical or cognitive impairments that compromise making appropriate use of the product are not intended to use Step Proactive.

Inclusion criteria to assess potential test participants were defined according to the characteristics of the intended users of the product. Therefore, for the assessment of the patient interface, participants were required to meet the following criteria: (1) have a cancer diagnosis; (2) be over 18 years old and under 75 years old; (3) understand and be able to communicate in Spanish; (4) have no prior familiarity with the application; (5) have the ability to use technology devices with internet access; and (6) have no physical or cognitive impairment that compromises making appropriate use of Step Proactive.

For HCPs, participants had to (1) understand and be able to communicate in Spanish, (2) have no prior experience with the application, and (3) be able to use an internet‐enabled technology device. In this case, participants had to (4) be HCPs specialized in oncology and compose a heterogeneous group with different careers (doctors and nurses) and sub‐specialties within oncology.

A total of 12 participants (six patients with a variety of cancer diagnoses and six HCPs) were recruited by Naru staff. All these participants were volunteers and met the specified inclusion criteria mentioned above.

Regarding the recruitment, the volunteers were offered the opportunity to participate in the test and provided their verbal consent. The consent process was facilitated through direct communication between the Naru team involved in the test and the participants, ensuring that each participant understood the test details and agreed to participate. The test ensured confidentiality and privacy; the participants' data was anonymized, and the usability test was not intended to introduce real data from each participant but instead conducted guided activities with the product. Participation was voluntary, and there was no compensation for participants in the test.

In the case of patients, all participants belonged to a non‐profit organization of cancer patients. The representatives of the organization were in charge of the diffusion of the invitations. A video was prepared by Naru explaining the following points: the objective of the sessions, the product, and the methodology to be followed. The representatives of the organization oversaw the diffusion of the video among the members and prepared a form where the data (diagnosis, age, relationship with the technology and contact) of the people interested in participating in the study were collected. This form was managed by the organization representatives to ensure the protection of the personal data and was never shared with any other entity. The non‐profit organization's representatives, who facilitated the recruitment process, were present and aware of the consent process, further ensuring that it was conducted ethically and transparently. Additionally, the test was not considered a risk for patients as it was carried out outside of their usual clinical routine and in a controlled environment.

This usability test did not require approval from the ethical committee as the purpose of the test is out of the scope of the regulations of clinical trials with medicinal products and medical devices [35], observational studies [36] and biomedical research [37]. The design of this study does not correspond to the investigation initiatives described as part of the definitions of these regulations since it does not involve the use of Step Proactive in accordance with its intended purpose in a real environment and for a continued period of time.

### 2.5 Sample Size

The question of how many participants are needed to effectively identify usability issues and gather valuable insights remains a topic of debate. While some studies advocate for larger participant numbers to enhance generalizability and statistical power, there is a compelling rationale for employing a smaller participant sample, around five to seven individuals.

Several studies have investigated the optimal participant number for usability testing. For instance, Nielsen and Landauer's seminal work on heuristic evaluation suggested that even a small group of five participants could uncover a substantial proportion of usability issues [38]. Furthermore, previous research [6, 22, 39, 40, 41, 42] has also conducted usability tests with a small number of participants (4–10) and has been able to identify a substantial number of usability issues.

In this study, the choice to involve a relatively small sample of six participants is based on several arguments. Smaller participant groups allow quicker data collection and efficient resource utilization. Given the constraints of time and resources in real‐world healthcare contexts, the selected size can give a reasonable result without compromising reliability.

Additionally, a smaller sample size can provide a more manageable and focused analysis of the user feedback, allowing for a more targeted approach in addressing usability issues [38, 43, 44, 45] mentioned that typically around five individuals have the potential to discover a significant proportion, approximately 80%, of usability errors or issues within a given system. Conducting usability testing with large numbers of participants can be time‐consuming in terms of time, personnel, and budget. The selected strategy of using a smaller set is in line with the efficient identification of major issues and implementing enhancements to improve the user experience, while also reducing cost, time, and resource investment.

Furthermore, Step Proactive has a previous generation device, Step Monitoring, with the same intended use and the same basic safety and performance characteristics, for which a previous cycle of usability testing was conducted. Issues identified during these usability tests were addressed as part of the improvements implemented in its successor product, Step Proactive.

Usability tests carried out in Step Proactive are a new iteration of the tests carried out with Step Monitoring. This new iteration is part of the Naru cyclic usability testing strategy. Evidence gathered from usability testing is used as an input for continuous product improvement. With the evidence on the usability tests of these six individuals per user profile, further usability evidence complementing the one obtained with Step Monitoring usability testing is gathered. Controlled and isolated changes were implemented into Step Monitoring interfaces during the development of Step Proactive. Therefore, a new group of six individuals per user profile is considered to be suitable to potentially detect 80% of usability issues associated with new product features and to increase the existing evidence of usability tests associated with features of Step Proactive that are common to Step Monitoring.

### 2.6 Performing Test

An appointment was arranged with each of the participants to perform the test. The testing session was held at Naru's facilities in order to conduct a single, face‐to‐face, one‐on‐one session. In all cases, participants used the devices provided by Naru for the execution of the activities related to the Step Proactive assessment. For each session, Naru staff members from the engineering and development teams were responsible for creating an account for each participant and providing the credentials to the participants. Furthermore, they were present in the sessions for technical support.

In addition to the participants, there were Naru personnel with different background profiles, who have extensive experience in conducting usability tests: researchers and quality assurance specialists. These profiles fulfilled the role of instructors and monitors of the test. These staff members have not been involved in the design and development of the Step Proactive product.

To avoid biased outcomes, participants were not provided with any additional information beyond that offered in the introductory session and collected in the data collection form for each session.

In order to gather valuable insights for product improvement, it is crucial that usability test sessions are effectively moderated. Instructors and monitors had to be familiar with the product field and have a clear understanding of the testing protocol. They have fluently guided the participants through the task and observed their interaction with the system interface. During this usability evaluation, Naru employees assumed the responsibilities of the instructors and monitors. They made the introduction session where they explained the purpose of the procedure and all scenarios and tasks. They observed and documented each user's interaction and noted the reactions and comments of each participant separately. All errors made by the user during task completion were recorded.

Before starting the test, each participant was asked to answer a pre‐questionnaire aimed at later analyzing the different profiles of participants in terms of representativeness of Step Proactive intended users. This questionnaire consists of four questions for HCP participants and six questions for patients' volunteers. The questionnaire addresses certain domains which are common to all the participants and include participants' gender, age, and technology skills measured as the extent to which participants use technology in their day‐to‐day activities. HCP participants were asked to provide their medical specialty in addition to the common domains of the pre‐questionnaire. Patient volunteers were asked to provide additional information regarding cancer type (primary tumor localization), whether or not they live accompanied, and whether or not they are dependent people.

The monitor asked participants to perform the relevant activities of each predefined scenario of the study. As usability testing takes place in a controlled environment, it was important for the monitor to simulate scenarios that provide context and purpose for the tasks being performed by the participant to reflect as much as possible real‐world situations. Additionally, each participant was asked to provide feedback aloud on the ease of completing each task or any challenges they faced during task performance, to capture and analyze tasks' difficulty expectations and perceptions. Following this protocol, participants verbalized what they saw, thought, did, and felt during the activities of each predefined scenario of the study, and their feedback was captured by the monitors at the test.

After each participant carried out all tasks, they then completed the SUS questionnaire [28]. Usability Metric for User Experience (UMUX) [46], Post‐Study System Usability Questionnaire (PSSUQ) [47] and the After Scenario Questionnaire (ASQ) [6, 48] are other commonly used instruments for measuring the usability of the application, but the SUS scale has been used and validated in a large number of studies over the years to assess perceived usability [49], which makes it highly reliable and allows it to be compared with other systems and applications. Its reliability and consistency have been statistically proved, achieving a Cronbach's Alpha 0.92 [49], showing consistency even for small sample sizes [50]. It has become the gold‐standard among Human Computer Interaction in the last years [51]. Achieving a large scale of perceived usability results across the health industry to support score understanding that other instruments cannot provide. Unlike more specific metrics (such as task time or error rate), SUS measures the user's overall satisfaction with the system. This provides a holistic view of the user experience, going beyond technical and performance details, capturing how the user feels about the product as a whole [52]. Some studies have shown that SUS has a high degree of reliability, validity, and can be adapted for different contexts [30, 53, 54, 55, 56, 57]. Thanks to being comprised of 10 questions, SUS allows for a quick but comprehensive evaluation of usability without requiring extensive efforts. This makes SUS an accessible and suitable tool, especially for iterative usability testing [52].

**FIGURE 5:**
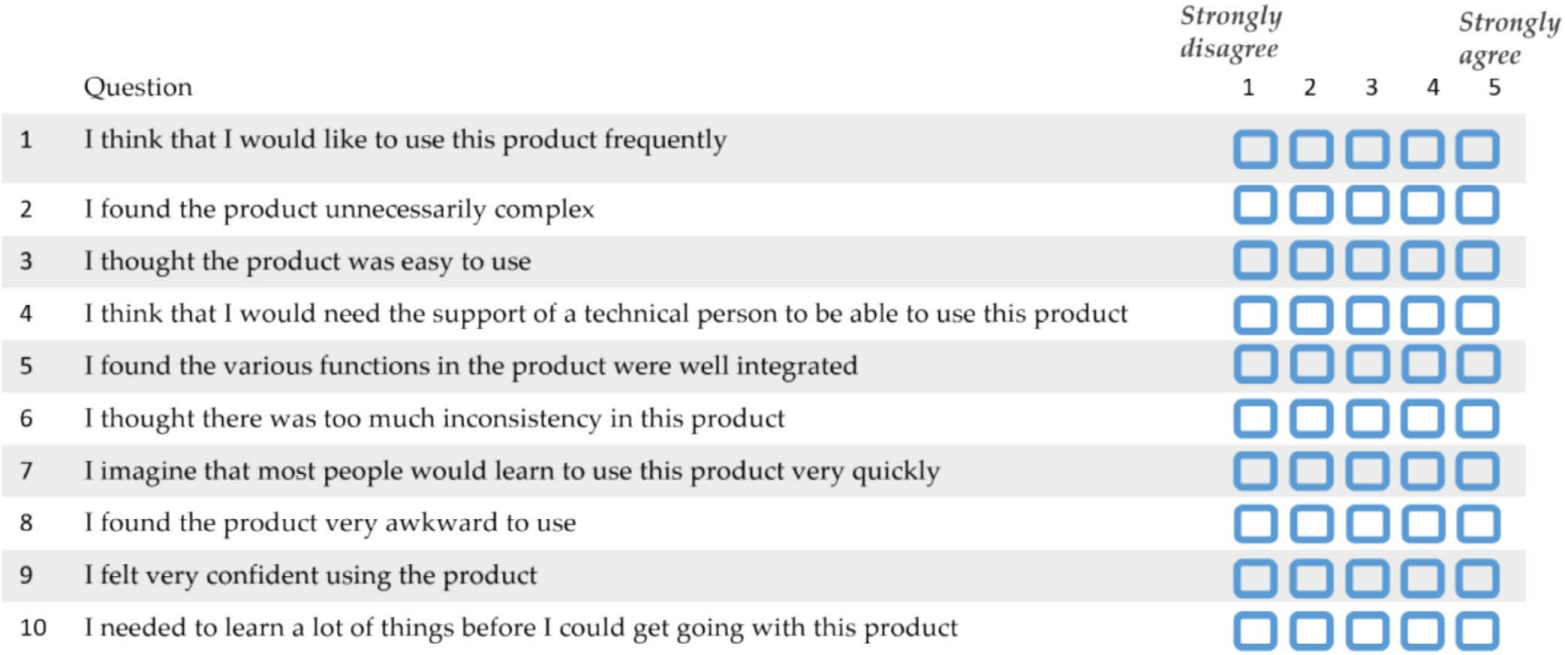
SUS questionnaire. The SUS questionnaire consists of a total of 10 items. The questions are divided into two categories: Usability‐related and learnability‐related. Usability related items, questions 1, 2, 3, 5, 6, 7, 8, and 9, focus on assessing the overall usability of the product service. Learnability‐related items, questions 4 and 10, evaluate how easily users can learn to use the product or service effectively.

The SUS consists of a set of 10 statements in total, half of them are positively toned (the odd number items), and the rest are negatively toned (the even number items). SUS items were selected by its developer J. Brooke [28] from a pool of 50 potential items. The 10 items that were included in the SUS were those that provided the strongest discrimination between a system that was relatively easy to use and another system that was relatively difficult [58]. Several studies have proved the reliability, validity, and sensitivity of the SUS [52, 58, 59, 60]. The SUS questionnaire is shown in Figure 5. The response is given on a scale of 1 (strongly disagree) to 5 (strongly agree) for each statement. The SUS score ranges from 0 to 100 (higher score meaning a better usability) in steps of 2.5 increments [51]. For positively worded items in the SUS, the score contribution is the scale position (scale goes from 1 to 5, 1 being “strongly disagree” and 5 being “strongly agree”) minus 1. For negatively worded items, it is 5 minus the scale position. To get the overall SUS score, the sum of the item score contributions shall be multiplied by 2.5 [58]. According to Lewis and Sauro et al. the SUS actually has two factors—usability (items 1, 2, 3, 5, 6, 7, 8, and 9) and Learnability (4 and 10) [61].

In this work, SUS scores were analyzed considering the overall SUS score and both the usability‐related and learnability‐related scores, as outlined by the authors in [62].

The work by Bangor et al. [63] considers the concept of applying a letter grade to the usability of the product as appealing because it is familiar to most of the people who work on design teams regardless of their discipline. Having an easy‐to‐understand, familiar reference point that can be easily understood by engineers and project managers facilitates the communication of the results of testing.

Several approaches, including absolute grading scales [63] and Curved Grading Scale (CGS) [62] have been proposed over the recent years; however, the CGS approach to a grading scheme is considered to be robust as it is based on data obtained during 30 years of usage from over 10,000 responses and hundreds of products [61]. This scale provides a good way to empirically interpret the meaning of the SUS scores.

**FIGURE 6:**
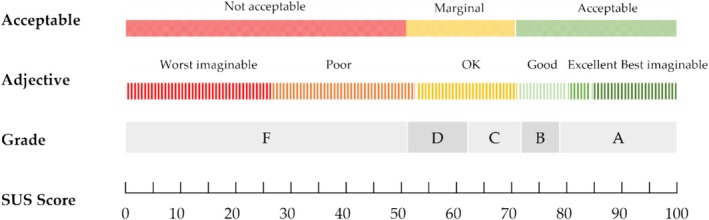
Interpretation of overall SUS scores. (1) Grades: From A (superior performance) to F (failing performance) according to the CGS for the SUS. (2) Adjective: From best imaginable (above 85) to worst imaginable (below 25). (3) Acceptable: acceptable (above 70), roughly acceptable (50–70) and not acceptable (below 50).

In this line, the overall SUS score can be interpreted in different ways [63], depending on the aim of the analysis, as represented in Figure 6: (1) Grades: the scores are categorized as grades, ranging from A, which indicates superior performance, to F, for failing performance, and grade C indicates an average performance, with the 50th percentile corresponding to a SUS overall score of 68 and located in grade C according to the CGS grading scheme (Table 2) [64]. CGS divides the top 15% of mean SUS scores into A+, A, and A−, and does a similar breakdown for B and C grades; however, providing similar distinctions for D and F grades does not seem as if it would be very useful (Table 2) [64]. (2) The adjectives are also used to express descriptions instead of numbers. In this case, the scores above 85 are associated with “Best Imaginable”, “Excellent” is for scores between 80 and 85, “Good” is just above the average, and “OK” is for scores from 51 to 70. (3) Another variation on using categories to describe the SUS is to think in terms of “acceptable” or “not acceptable”. Acceptable scores fall above 70, while scores below 50 belong to the unacceptable classification. The range between 50 and 70 is referred to as “marginal” [61]. It is becoming a common industrial goal to achieve a SUS of 80 as evidence of an above‐average user experience, which seems a reasonable benchmark given that it is a B in the proposed absolute grading scale [63] and an A− in the CGS [64].

**TABLE 2:** Curved Grading Scale for the System Usability Scale [64].

Grade	SUS	Percentile range
A+	84.1–100	96–100
A	80.8–84.0	90–95
A−	78.9–80.7	85–89
B+	77.2–78.8	80–84
B	74.1–77.1	70–79
B−	72.6–74.0	65–69
C+	71.1–72.5	60–64
C	65.0–71.0	41–59
C−	62.7–64.9	35–40
D	51.7–62.6	15–34
F	0–51.6	0–14

Median overall SUS scores obtained for each user profile during usability testing are matched to the corresponding CGS grade, adjective, and acceptability score in Figure 6 to facilitate the interpretation of the results of thetesting.

### 2.7 Analyzing the Results

This stage includes gathering and analyzing all the information of the usability test data to identify critical and actionable insights on how to improve Step Proactive usability. No missing values were found in the evaluation variables; therefore, no data processing techniques were required, and the complete sample was used to conduct the analysis.

The results were analyzed in a comprehensive manner, including (1) a descriptive summary of the demographic data collected through the questionnaire, (2) scenario completion rates according to the pass/fail criteria, and (3) scores from the SUS questionnaires.

Scenario and activities completion rates were evaluated as frequencies and percentages. Scenarios and activities with a low pass ratio arise as critical aspects on which a thorough assessment should be carried out. Safety‐related activities are expected to achieve a 100% completion rate in order to not be labeled as a critical area for improvement. SUS scores were reported with median and interquartile range (IQR). Unacceptable SUS scores shall mark a course of action required to address the need to improve the ease of use of the tool. Demographic data collected was used to understand result patterns and potential limitations due to differences in participants' demographic profiles.

Furthermore, all the user qualitative feedback was analyzed to identify comments and improvements, combining both deductive and inductive methodologies conducted by two different researchers. First, preliminary categories were defined to maintain alignment with the research questions as part of the deductive approach. Then, each researcher independently labeled feedback into the relevant categories. In case of no matching category being found, an inductive approach was adopted to propose a new category or expand an existing one. The two resulting categorizations were then discussed until an agreement was reached. This was done for both interfaces of Step Pro‐active, patients and HCPs. Finally, all the results were organized in order to identify patterns, synthesize findings, and determine actionable recommendations.

## 3 Results

### 3.1 Participants

The analysis of the user demographic shows the results of the 12 participants (six patients and six HCPs) involved in the usability test of Step Proactive and their correspondence with Step Proactive's intended user characteristics.

Six volunteers who have at some point been diagnosed with cancer, aged between 32 and 72 years, were recruited for the usability evaluation of the patient interface of Step Proactive. The distribution of participants was such that 83.3% were women, while men contributed 16.7% of the total participants. The average age was 47.8 years, and 100% of the participants reported using different technologies, namely computers, cell phones, and/or tablets, 83.3% indiscriminately in their daily activities, both at work and in their lives, while 16.7% reported using technology only at work and/or for specific day‐to‐day situations.

66.6% of the participants have or had breast cancer, 16.7% had squamous cell carcinoma, and another 16.7% had prostate cancer. 100% of the participants confirmed that they were not dependent, and 66.6% stated that they live in company, while 33.4% live alone.

Six participants in the usability assessment of the HCPs interface of Step Proactive were HCPs with experience in oncology services. The distribution of participants was such that 66.6% were women and 33.4% were men. The mean age was 30.2, and 100% of the participants reported using different technologies, specifically, computers, cell phones, and/or tablets interchangeably in their daily activities, both at work and in their personal lives. 50% of the participants were medical oncologists, 16.7% were hematologist‐oncologists, 16.7% of the participants were nurses, and 16.7% had a profile of a physician from the radiation oncology service.

### 3.2 Scenario Completion Rates

Usability refers to how well an intended user can use a system to achieve its intended purpose and how stable the system is to user errors and potential risks. In order to assess usability among patients and HCPs in this aspect, tests were conducted using various scenarios in face‐to‐face sessions.

A total of 16 activities for patient interface of Step Proactive were evaluated according to the pass/fail criteria. The acceptance criteria for this evaluation required successful completion of all activities pertaining to product safety and evaluation of ease of use. All participants have understood and completed correctly 100% of the activities related to product safety and ease of use. Only two of six participants made an error in the log‐in activities. However, it is important to clarify that this error is not related to the security of the product. Considering 66.6% of the participants have completed the activity correctly, no mitigation measures have been considered to be implemented.

For the evaluation of the HCP interface of Step Proactive, a total of 36 activities were evaluated with the same acceptance criteria. All the participants understood and completed 100% of the activities correctly.

### 3.3 SUS Questionnaires Scores

Additionally, to usability evaluation with scenarios, participants completed the SUS questionnaire Figure 5. The SUS approach helps to measure the usability as the capacity of the product to be effectively used by specific users in order to achieve specific objectives with efficiency and satisfaction in an objective manner. This method consists of measuring the usability of the tool through 10 questions, rated between 1 and 5 (1—strongly disagree, 2—disagree, 3—neutral, 4—agree, 5—strongly agree) to obtain the corresponding SUS score. The SUS provides a summary score between 0 (indicating poor usability) and 100 (representing excellent usability).

To align the interpretation of results and facilitate the comparison of all responses within a standardized framework, a transformation is applied to the negatively worded questions. Solely on the visualization part of results, even items of the SUS questionnaire are rewritten with a positive tone. Rewriting of the SUS questionnaire even items with a positive tone was first proposed by Sauro and Lewis and proved to have no significant effect on the SUS scores [49]. In this case, considering that participants responded to the original SUS questionnaire, the original scoring system was followed. Rewriting of even items to a positive tone for results visualization ensures a negative connotation for the “Strongly disagree” and “Disagree” terms across SUS items, and a positive connotation for the “Strongly agree” and “Agree” terms. In this sense, participants' original responses to even items were converted for results visualization purposes: “Strongly disagree” values were converted to “Strongly agree” values and vice versa, and “Disagree” values were converted to “Agree” values and vice versa.

**FIGURE 7:**
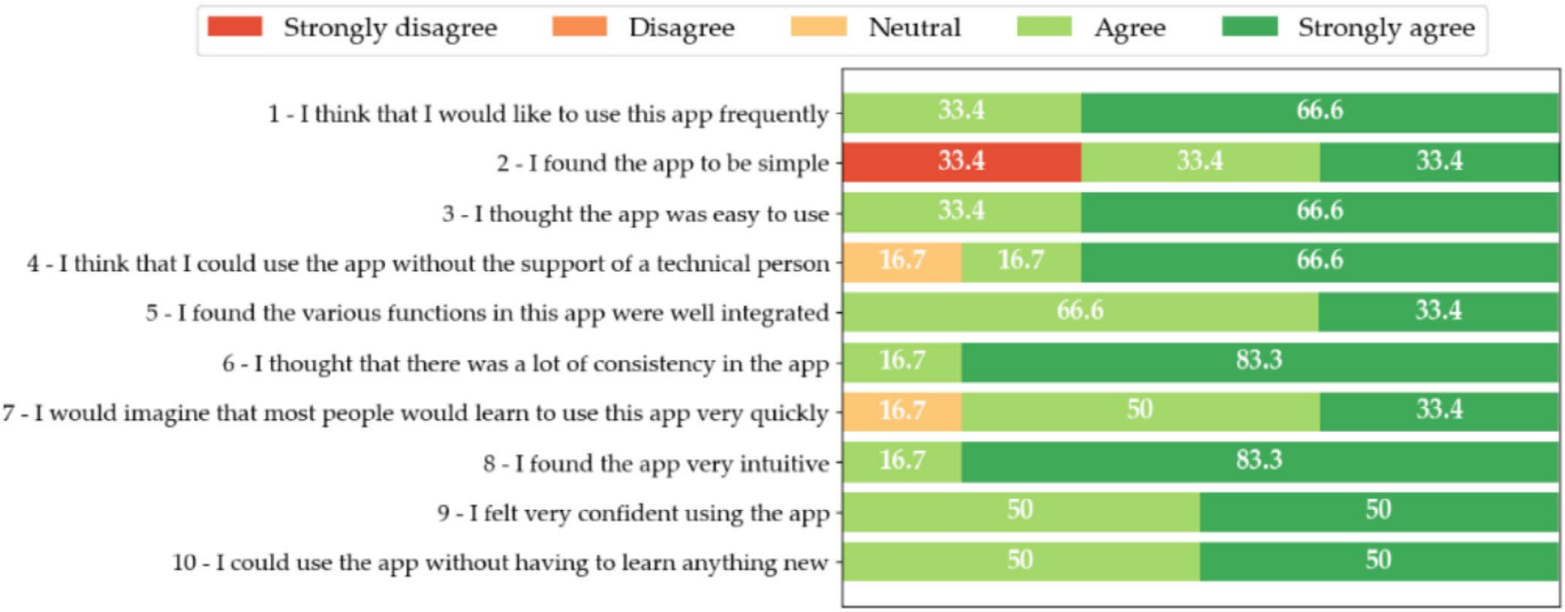
SUS results of the usability evaluation of step proactive. Percentages of SUS item responses for step proactive's patient interface.

**FIGURE 8:**
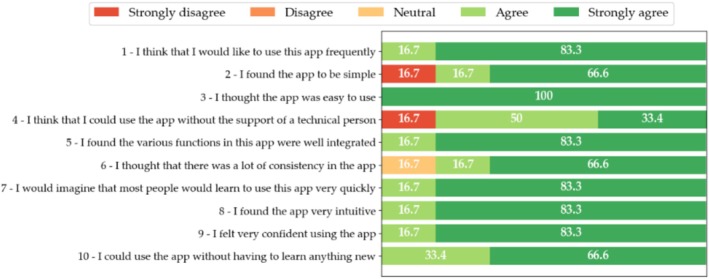
SUS results of the usability evaluation of step proactive. Percentages of SUS item responses for step proactive's HCP interface.

The usability study of both the patient and HCP interfaces of Step Proactive comprised six participants for each interface who all successfully navigated the SUS. Results are shown in Figures 7 and 8. The figures represent the percentages of each response obtained from SUS items.

In the case of the patient interface evaluation of Step Proactive, 7 of the 10 items were rated with a score of 4 or 5 out of 5 by all participants. 83.3% believe that most people would learn to use the application very quickly (SUS item 7) and that the application is very consistent (SUS item 6). However, 33.4% of the participants consider the app to be unnecessarily complex (SUS item 2).

In the case of Step Proactive's HCP interface, as in the previous case, 7 out of 10 items were evaluated with a score greater than or equal to 4 out of 5. Furthermore, 6 out of 10 items have been evaluated with the optimal score (5 out of 5) by more than 83.3% of the participants. In this case, 100% of the participants believe that the application is easy to use (SUS item 3). However, 16.7% of the participants consider the application to be unnecessarily complex (SUS item 2) and believe that they would need help from staff (SUS item 4).

**FIGURE 9:**
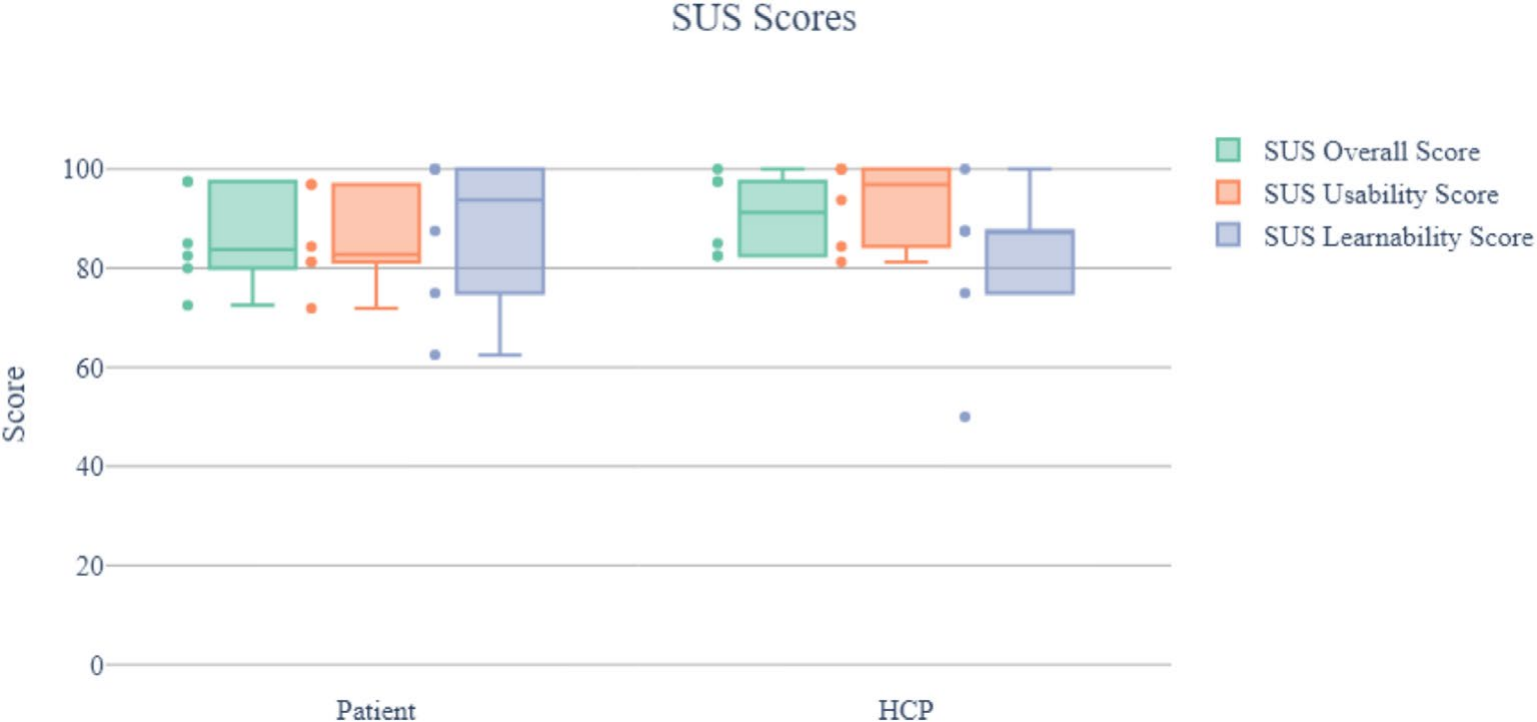
SUS scores. The figure represents the SUS scores, usability‐related scores, and learnability‐related scores obtained for both the patient and HCP interfaces evaluation of step proactive.

The median (IQR) overall SUS scores were 83.75 (80.62–94.37) and 91.25 (83.12–97.50) for the patient and HCP versions of the Step Proactive product, respectively. Although SUS was originally designed to assess perceived usability as a single attribute, Lewis and Sauro [65] found that there are actually two factors in SUS—usability (related to 8 items) and Learnability (related to 2 items). Figure 9 shows the results obtained for each interface.

As can be seen, all median scores have a value higher than 80 for both interfaces.

In the case of the patient interface, the median (IQR) learnability score was 93.75 (78.12–100), a slightly higher value than the overall SUS and usability values, which were 83.75 (80.62–94.37) and 82.81 (81.25–93.75) respectively. On the other hand, the HCP interface shows higher overall median SUS and usability values, 91.25 (83.12–97.50) and 96.87 (86.72–100), higher than learnability, which was 87.50 (78.12–87.50).

Overall, SUS and usability values follow a similar pattern and have a homogeneous distribution across both interfaces. However, the learnability values are more dispersed. Furthermore, the overall SUS grade in both interfaces is “A” A+ for HCP interface and A for patient interface according to Table 2, which corresponds to the adjective “Best Imaginable” for HCP interface and “Excellent” for patient interface, and the product is acceptable according to the acceptability criteria as it is shown in Figure 6.

Based on the grading system in Figure 6, both interfaces achieved a superior performance with “A” rating. Using qualitative descriptions, the usability of both interfaces can be described as “Acceptable” and “Best Imaginable” for the HCP interface and “Excellent” for the patient interface.

### 3.4 Qualitative Participants Feedback

In addition to analyzing the scenario and SUS completion, we examined all qualitative feedback provided by users to identify comments and potential areas of improvement. Regarding the qualitative feedback of the participants, most of them indicated that the interface was intuitive since it resembled the operation of the applications they usually use. Furthermore, several participants remarked that they would use the application in daily routines and others stated that they would recommend the application to other users.

Finally, all the results were organized in order to identify patterns, synthesize findings, and determine actionable recommendations. Notably, none of them were related to product safety relevant activities.

As a part of Naru's iterative usability testing as a continuous product improvement tool, potential improvements that impact the most user experience and safety are focused on. With a strong emphasis on this approach, results were analyzed, and it was acknowledged that most identified issues could be addressed by enhancing the accessibility of tutorials.

## 4 Discussion

### 4.1 Principal Findings

The principal aim of this study was to objectively measure the usability and user‐friendliness of Step Proactive and, secondly, to collect feedback from potential users regarding the use of Step Proactive, which was the device under investigation. The study achieved its goals effectively by providing valuable insights into user experience in the different categories.

To this end, the work includes evaluation sessions with users of different backgrounds, both patients and HCPs. In total, six volunteer participants took part in the test for each Step Proactive interface. A number which, according to the articles published by [38, 43, 44] is sufficient, as it is considered that with five participants, 80% of the possible errors can be identified. A detailed plan with the protocol of the sessions was defined to collect both quantitative and qualitative data through feedback from the participants, and the participants had to carry out the activities previously defined in the plan.

In general, 100% of all participants successfully completed the activities of the usability test. This finding showed that the tool was found easy to use. However, during the session, an issue related to a single‐use error in the application login process was observed in the patient interface of Step Proactive. It's important to note that this error doesn't compromise the security of the product. On the contrary, 100% of Step Proactive's safety‐related activities were carried out correctly, demonstrating the tool's ease of use. In addition, [34, 66] researchers have shown that if end‐users are familiar with using a device over a long period of time, then familiarity translates into a perception of increased usability. However, one of the participants was not familiar with smartphone applications but managed to complete 100% of the activities correctly.

In order to measure the usability of Step Proactive in an objective manner, the SUS questionnaire was used. The median (IQR) SUS overall scores were 83.75 (80.62–94.37) and 91.25 (83.12–97.50) for the patient and HCP interfaces of Step Proactive, respectively. Regarding the CGS grading scheme, these results qualify the Step Proactive product as graded A for patients and A+ for HCPs (Table 2), indicating superior performance of the product and overcoming the industrial goal of 80 as evidence of above‐average user experience. Furthermore, the adjectives that correspond to Step Proactive are Excellent for the patients' interface and Best Imaginable for HCPs' interface, which are the two upper values of the 7‐point adjective scale and are ranked as acceptable in the acceptability approach (Figure 6). Since usability is defined as the capacity of the product to be effectively used by specific users in order to achieve specific objectives with efficiency and satisfaction, the obtained results may infer user satisfaction when using Step Proactive.

According to recent studies, the SUS scale measures both usability and learnability [65]. For the patient interface, the three attributes (overall SUS, usability, and learnability) have higher values than 85. For the HCP interface, the learnability attribute has more dispersed values and a lower value than the other two attributes (overall SUS and usability). It was concluded that there is an outlier in the learnability attribute.

After analyzing the results of the SUS questionnaire, it was found that there might exist a misinterpretation or data entry inconsistency in the SUS question number 4 (I think that I would need the support of a technical person to be able to use this product) and number 2 (I found the product unnecessarily complex). A potential question misinterpretation or human error in data entry is suspected in both questions for two reasons: (1) same patient's opposite answers to questions with similar statements but different wording in the usability testing and (2) same patient's positive qualitative feedback on the same topic as the questions.

Additionally, even if the learnability attribute has lower values for the HCP interface, HCP users in a real environment will have a training session of Step Proactive interfaces before using it in their daily work. It is a contraindication of the Step Proactive HCP interface that HCP users who have not been trained shall not use it.

The qualitative results reinforced aspects identified through the quantitative evaluation. Qualitative results were positive, concluding that Step Proactive is found by users to be an ease of use and a useful tool. The intuitiveness of the interface was remarked upon, and potential users stated that they would like to use the application on a daily basis.

Evidence gathered from user feedback provided improvement opportunities in Step Proactive. In this sense, the tutorial of the application was made accessible not only the first time using the application but also anytime during its use. This will allow users to easily repeat the tutorial whenever they want, thereby facilitating the use of the tool. Also, including this feature can help to avoid possible use errors, since patients will be able to review the most relevant functionalities of the application whenever they want.

### 4.2 Limitation

One of the main study limitations is the number of languages in which the study was carried out. Also, participants who acknowledged avoiding the use of technologies could not be recruited. Although the probability of this population using Step Proactive in a real‐world scenario could be very low, it would have been of great interest to be able to assess Step Proactive with volunteers reluctant to use such technologies. Even though the number of usability test participants is considered sufficient and approximately 80% of usability errors or issues are identified [38, 43, 44, 45], an improvement opportunity was identified to increase sex, age range, diversity in user comfort with technology, and even geographical diversity (including language diversity) as well as the capacity to identify more usability issues by gathering a bigger participant sample. The planned future work will address this limitation as further study is planned as a next step in a clinical setting where usability will be measured in a real environment use of Step Proactive.

## 5 Conclusion

This usability test of both interfaces of the Step Proactive clinical decision support system for remote monitoring of AEs of patients with cancer demonstrates that all participants have understood and completed 100% of the activities correctly. Furthermore, it can be concluded from the results that the participants found the Step Proactive interfaces easy to use, with a clear and straightforward design, and found it useful to better cope with AEs and to improve clinical follow‐up of patients with cancer during and after cancer treatment. Regarding the SUS grading, both interfaces were rated as grade A (A+ for HCPs' interface and A for the patients' interface considering the CGS breakdown for A grades) with the adjective of “Best Imaginable” for HCPs' interface and “Excellent” for patients' interface, indicating the superior performance of the product.

Furthermore, it enables the identification of actionable recommendations. Adopting some of the additional measures identified from the suggestions made by the participants during the sessions has been considered. These improvements do not affect either its intended functionality or the overall functioning of the tool. However, they help to avoid potential use errors and lead to some developments to refine Step Proactive interfaces.

The planned future work starts by implementing the improvements detected during the usability test in Step. Proactive interfaces and further studies on usability in a clinical setting are needed.

## Author Contributions

**D.C., M.A., A.A., M.S., and M.A.I.:** methodology. **D.C., M.A., A.A., and M.S.:** analyzed the data. **D.C. and M.A.:** writing original draft preparation. **D.C., M.A., M.E., A.A., M.S., R.Á., and M.A.I.:** writing – review and editing. All authors reviewed and approved the final manuscript.

## Ethics Statement

Human Ethics and Consent to Participate declarations: not applicable. This usability study involves user interaction with technology, incorporating observation and feedback sessions with participants. It was conducted in a controlled environment with minimal risk for participants. Thus, this study falls outside the scope of formal ethical committee approval requirements. All participants were informed about the purpose of the study, and they provided their verbal consent to participate. The study ensured confidentiality and privacy; the participants data were anonymized. Participation was voluntary and there was no compensation for patients and clinicians in the study.

## Conflicts of Interest

All authors are employees of Naru, and M.A.I. owns stock in Naru.

## Data Availability

The datasets generated and analyzed during this study are not publicly available to ensure participant privacy. Deidentified outcome datasets can be obtained from the corresponding author upon reasonable request.
